# Sir James Y. Simpson on Medical Progress

**Published:** 1868-12

**Authors:** 


### Miscellaneous.

### Sir James Y. Simpson on Medical Progress.

At the close of the ceremony of "capping" the medical graduates of the University of Edinburgh, recently, Sir James Simpson delivered an address. In the course of his remarks, he said,—"A most extensive field for new investigations lies temptingly open for the young and ambitious physician in the almost innumerable series of new chymical compounds which modern organic chymistry has solved. Among this world of new compounds will probably be yet detected therapeutic agents more direct, more swift, and yet more sure in their action than any which our present pharmacopœias can boast of. It may be, also, that the day will yet come when our patients will be asked to breathe or inspire most of their drugs instead of swallowing them; or at least when they will be changed into pleasant beverages instead of disgusting draughts and powders, boluses and pills. But that day of revolution will not probably be fully realized till those distant days when physicians—a century or two hence—shall be familiar with the chymistry of most diseases; when they shall know the exact organic poisons which produce them, with all their exact antidotes and eliminatories; when they shall look upon the cure of some maladies as simply a series of chymical problems and formulæ; when they shall melt down all calculi, necrosed bones, etc., chymically, and not remove them by surgical operations; when the bleeding in amputations and other wounds shall be stemmed, not by septic ligatures or stupid needles, but by the simple application of hæmostic gases or washes; when the few wounds then required in surgery shall all be swiftly and immediately healed by the first intention; when medical men shall be able to stay the ravages of tubercle, blot out fevers and inflammations, avert and melt down morbid growths, cure cancer, destroy all morbific organic germs and ferments, annul the deadly influences of malaria and contagions, and by those and various other means markedly lengthen out the average duration of human life; when our hygienic condition and laws shall have been changed by State legislation, so as to forbid all communicable diseases from being communicated, and remove all causes of sickness that are removable; when the rapidly increasing length of human life shall begin to fulfill that ancient prophecy, "the child shall die an hundred years old;"—when there shall have been achieved, too, advances in other walks of life far beyond our present state of progress; when houses shall be built and many other kinds of work performed by machinery, and not by human hands alone; when the crops in these islands shall be increased five or ten fold, and abundance of human food be provided for our increased population by our fields being irrigated by that waste organic refuse of our towns which we now recklessly run off into our rivers and seas; when man shall have invented means of calling down rain at will; when he shall have gained cheaper and better motive power than steam; when he shall travel from continent to continent by submarine railways, or by flying and ballooning through the air; and when—to venture on only one illustration more—tiresome graduation addresses shall no longer require to be written by old professors nor listened to by young physicians."—*California Med. Gazette.*

Remedy for Toothache.—Alum, reduced to an impalpable powder, two drahms; nitrous spirits of ether, seven drahms; mix and apply to the tooth.

